# Development and validation of a measure of supervisory best practices in gerontological social work

**DOI:** 10.1093/geroni/igab046.3379

**Published:** 2021-12-17

**Authors:** Daniel Kaplan, Keith Chan

**Affiliations:** 1 Adelphi University, Cortlandt Manor, New York, United States; 2 Hunter College, New York, New York, United States

## Abstract

Agency-based supervision is essential to skilled practice and staff retention, directly impacting the quality of services. The Supervisory Leaders in Aging (SLA) program was designed to strengthen supervision of the aging services workforce. SLA was implemented in four states and trained 134 social work supervisors. The purpose of this study was to develop a novel, comprehensive, and practice-informed measure of supervisory best-practices in gerontological social services. The primary outcome for the SLA program was the Practice Inventory for Supervision in Aging Services (PISAS), which evaluated the frequency of recent use of best practices as identified by the project team and vetted by instructors and an interdisciplinary advisory board. Reliability and confirmatory factor analyses were conducted to examine its psychometric properties. Findings demonstrated the scale had good internal consistency reliability (ɑ = 0.88). Confirmatory factor analyses indicated a three-factor solution, 1) Gerontological Social Work Skills, 2) Program Development Skills, and 3) Supervision and Leadership Skills, accounting for 72% of the variability in the 27 items. Gerontological Social Work Skills captured best practices regarding mental health in late life, heightening awareness of elder abuse, and working with families. Program Development Skills captured assessment, measuring outcomes, and translating evidence into practice. Supervision and Leadership Skills captured individual supervision, group supervision and leadership in interdisciplinary practice. Results indicated that PISAS is a reliable and valid measure of use of best practices in gerontological social work supervision. Further implications and limitations of this measure in assessing outcomes of gerontological education and training programs are discussed.

